# Characterization of a monkey model with experimental retinal damage induced by N-methyl-D-aspartate

**DOI:** 10.1242/dmm.050033

**Published:** 2024-07-26

**Authors:** Guo Liu, Longxiang Huang, Junkai Tan, Yun Wang, Chunlin Lan, Yaxi Chen, Yukai Mao, Xizhen Wang, Ning Fan, Yihua Zhu, Xianjun Zhu, Xuyang Liu

**Affiliations:** ^1^ Sichuan Provincial Key Laboratory for Human Disease Gene Study, Sichuan Provincial People's Hospital, University of Electronic Science and Technology of China, Chengdu, 610072, China; ^2^ The First Affiliated Hospital of Fujian Medical University, Fuzhou, Fujian, 350005, China; ^3^ Xiamen Eye Center, Xiamen University, Xiamen, 361004, China; ^4^ Shenzhen Key Laboratory of Ophthalmology, Shenzhen Eye Hospital, Jinan University, Shenzhen, 518040, China; ^5^ Research Unit for Blindness Prevention of Chinese Academy of Medical Sciences (2019RU026), Sichuan Academy of Medical Sciences & Sichuan Provincial People's Hospital, Chengdu, Sichuan, 610072, China; ^6^ Department of Ophthalmology, Shenzhen People's Hospital, the 2nd Clinical Medical College, Jinan University, Shenzhen, 518020, China

**Keywords:** Monkey model, NMDA, Retinal damage, Optical coherence tomography, Electroretinography

## Abstract

N-methyl-D-aspartate (NMDA)-induced retinal damage has been well studied in rodents, but the detailed mechanisms have not yet been characterized in nonhuman primates. Here, we characterized the retinal degenerative effects of NMDA on rhesus monkeys *in vivo*. NMDA saline or saline-only control was injected intravitreally to the randomly assigned eyes and contralateral eyes of four rhesus monkeys, respectively. The structural and functional changes of retina were characterized by optical coherence tomography and electroretinography on days 0, 4, 30 and 60 post injection. Both optic discs and macular areas of the NMDA-injected eyes initially presented with a transient retinal thickening, followed by continued retinal thinning. The initial, transient retinal thickening has also been observed in glaucoma patients, but this has not been reported in rodent NMDA models. This initial response was followed by loss of retina ganglion cells (RGCs), which is similar to glaucomatous optic neuropathy and other RGC-related retinal degenerations. The amplitudes of both the photopic negative response and pattern electroretinogram decreased significantly and remained low until the end of the study. Thus, the NMDA monkey model may serve as a more clinically relevant animal model of retinal damage.

## Teaser

**Summary:** Retinal damage was experimentally induced in rhesus monkeys by intravitreal injection of N-methyl-D-aspartate (NMDA). Optical coherence tomography and electroretinography examinations showed similarities with human retinal diseases.

## INTRODUCTION

Screening for potential neuroprotective agents has become more and more attractive in the ophthalmic pharmaceutical research area, especially for glaucoma and retinal degenerating diseases in recent years (Gokoffski et al., 2020). N-methyl-D-aspartate (NMDA)-induced retinal damage is an adequately developed retinal injury model in rodents, in which the mechanisms have been demonstrated relatively well (Honda et al., 2019; Yan et al.,2020). Glutamate, an excitatory amino acid neurotransmitter, plays an important role in synaptic signal transduction and is required in early retinal development (Kaur et al., 2012). It stimulates neuronal glutamate receptors, including NMDA receptors. NMDA receptors are a family of ionotropic glutamate receptors, responsible for synaptic excitations (Crook et al., 2014). However, under some abnormal circumstances, such as the hypoxia-ischemia condition, elevated levels of glutamate have been detected. Excessive glutamate can cause excitotoxicity to retinal ganglion cells (RGCs), mainly mediated by the NMDA receptors, and lead to selective RGC apoptosis (Hof et al., 1998; Ivanova et al., 2020; Kaur et al., 2012). In rodents, intravitreal injection of glutamate or its analog, NMDA, induces RGC death. Hence, NMDA-induced retinopathy in rats, mice and rabbits has been used as a model for retinal diseases, such as glaucoma, retinal ischemia and diabetic retinopathy (Kinoshita et al., 2020; Niwa et al., 2016). However, to our knowledge, the detailed mechanisms of NMDA-induced retinal damage in monkeys have not been characterized yet.

Nonhuman primate models, especially macaque models, are essential for drug research and development because of their close similarities in physiology, pathology, metabolism, biochemistry and genetics to those of human beings (Shen, 2010). Nonhuman primate models are a critical translational bridge between preclinical studies in rodent models and human clinical studies (Kleinert et al., 2018). However, due to their long lifespan, low breeding rate, relatively high cost, difficulty in procurement and strict husbandry requirement, the application of nonhuman primate models is very limited (Kleinert et al., 2018). The NMDA-induced retinal injury model has been well studied in rodents, but has not been well characterized in macaques. Only limited reports have been published (Cohen and Miller, 1994; Hood et al., 1999; Rangaswamy et al., 2004, 2003; Ueno et al., 2004, 2006; Viswanathan et al., 2002). To our knowledge, the current study is the first that characterizes the detailed structural and functional changes seen in NMDA-induced retinal damage in monkeys.

## RESULTS

**Fig. 1.**
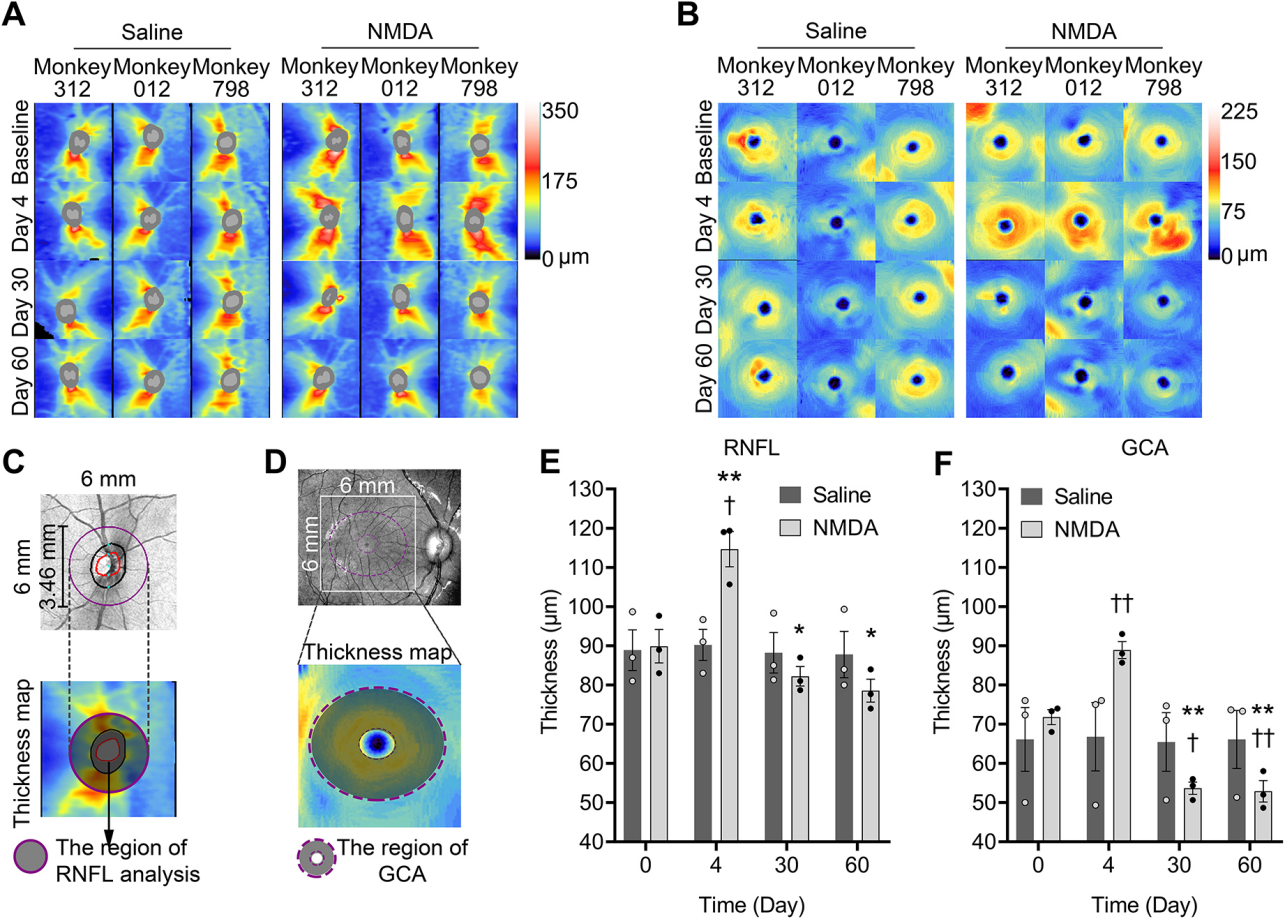
**Thickness changes in the circum-papillary retinal nerve fiber layer and macular ganglion cell analysis after intravitreal injection of NMDA and saline.** Monkeys 312, 012 and 798 were used for these analyses. (A) Thickness maps in the retinal nerve fiber layer (RNFL). (B) Thickness maps in ganglion cell analysis (GCA). (C) Schematic diagrams of RNFL thickness analysis. Purple circles mark the circum-papillary region, and the black and red regions represent the optic disc margin and the optic cup margin, respectively. (D) Schematic diagrams of thickness analysis by GCA. The regions between the outer and inner dashed purple ellipses indicate the region analyzed. (E) Time courses of RNFL thickness changes. Repeated-measures two-way ANOVA detected significance in groups and time interaction (*F*=37.03, *P*<0.0001), time factor (*F*=40.01, *P*<0.0001) and subjects factor (*F*=22.17, *P*<0.0001). The group factor (*F*=1.164, *P*=0.6862) was not significant. (F) Time courses of thickness changes by GCA. Repeated-measures two-way ANOVA detected significance on groups and time interaction (*F*=94.86, *P*<0.0001), time factor (*F*=105.5, *P*<0.0001) and subjects factor (*F*=89.52, *P*<0.0001) of GCA results. The group factor (*F*=0.0074, *P*=0.9357) was not significant. Post hoc analysis was conducted when the effects of group, time, or the interaction between group and time were significant. *P<0.05 and **P<0.01 between the NMDA and saline groups; ^†^*P*<0.05 and ^††^*P*<0.01 versus day 0 by Fisher's least significant difference (LSD) post hoc test. Data are shown as mean±s.e.m. (*n*=3).

Optical coherence tomography (OCT) assessment indicated that the thicknesses of the circum-papillary inner retinal layers – the retinal nerve fiber layer (RNFL), and the ganglion cell layer (GCL) along with the inner plexiform layer (IPL) [measured by the ganglion cell analysis (GCA) algorithm] – were not changed in the control group after saline injection at all time points evaluated (Fig. 1). In contrast, in the NMDA-injected eyes, both the RNFL and the GCL+IPL increased significantly in thickness at day 4, followed by thinning in the subsequent days (Fig. 1).

### Thickness changes of circum-papillary and macular RNFL

The RNFL thickness heat maps of the circum-papillary areas are shown in Fig. 1A, and the corresponding RNFL thickness column charts are shown in Fig. 1E. The shaded area in Fig. 1C marks the region measured in RNFL analysis. The baseline thickness (day 0) averaged 89.89±4.26 μm (mean±s.e.m.) and 88.89±5.19 μm in the eyes before intravitreal injections with NMDA and saline, respectively (*n*=3). Repeated-measures two-way ANOVA detected significance of groups and time interaction (*F*=37.03, *P*<0.0001), time factor (*F*=40.01, *P*<0.0001) and subjects factor (*F*=22.17, *P*<0.0001). Only the group factor (*F*=1.164, *P*=0.6862) was not significant. Fisher's least significant difference (LSD) post hoc test indicated that there was no statistical difference in the baseline thickness between the two groups (*P*>0.05). In the saline-injected group, no significance was detected when compared to baseline (*P*>0.05). The NMDA-injected eyes showed RNFL thickening at day 4 (114.67±4.50 μm; *P*<0.01 versus saline-injected eyes; *P*<0.05 versus day 0), and then gradual thinning with time, even though the differences were not statistically significant (82.22±2.48 μm on day 30, *P*=0.0528 versus day 0; 78.56±2.92 μm on day 60, *P*=0.0644 versus day 0). However, the mean difference of RNFL thickness between bilateral eyes on day 30 or day 60 was significantly different (−6.00±2.69 μm on day 30, *P*<0.05 versus control; −9.22±3.77 μm on day 60, *P*<0.05 versus control; negative values indicate the decrease in thickness), which also indicated that the RNFL thinning was induced by NMDA.

### Thickness changes of macular areas by GCA

The GCA algorithm measures the thickness of GCL+IPL. The GCA maps of the macular areas are displayed in Fig. 1B, and corresponding GCA thickness column charts are shown in Fig. 1F. The shaded area in Fig. 1D indicates the region measured region for GCA. The baseline thickness was 71.78±1.90 μm and 66.11±8.12 μm in eyes to be injected intravitreally with NMDA and saline, respectively (*n*=3 per group). Repeated-measures two-way ANOVA detected significant effects of time (*F*=105.5, *P*<0.0001), the interaction between group and time (*F*=94.86, *P*<0.0001) and the individual subject (*F*=89.52, *P*<0.0001). Only the group factor (*F*=0.0074, *P*=0.9357) was not significant. GCA with Fisher's LSD post hoc test did not show significant changes in the thickness between post-treatment and baseline groups (*P*>0.05). Similar to the changes in circum-papillary RNFL thickness, GCA also showed an initial increase (88.89±2.16 μm at day 4; *P*<0.01 versus day 0) and then a significant decrease in the NMDA group (53.67±1.58 μm on day 30, *P*<0.05 versus day 0; 52.89±2.73 μm on day 60, *P*<0.01 versus day 0). Furthermore, the mean difference in thickness measured by GCA between bilateral eyes on day 30 or day 60 was significantly different (−11.78±8.15 μm on day 30, *P*<0.01 versus control; −13.22±5.59 μm on day 60, *P*<0.01 versus control).

### Changes in optical nerve head parameters

**Fig. 2.**
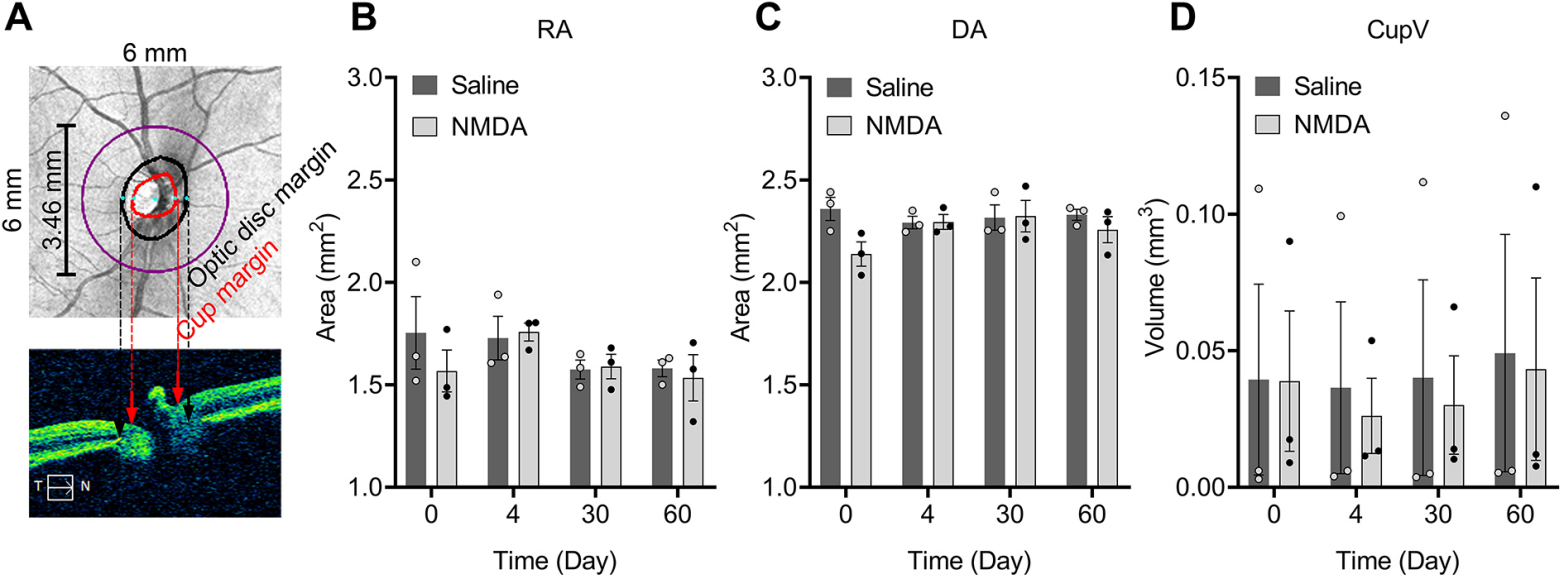
**Changes to optical nerve head parameters after intravitreal injection of NMDA.** Monkeys 312, 012 and 798 were used for these analyses. (A) Schematic diagram showing the regions measured for relative parameters including rim area (RA), disc area (DA) and cup volume (CupV) on the optical nerve head. Red dashed arrows indicate the cup margins on the horizontal tomogram (bottom panel) corresponding to that on the deviation map (upper panel). Black dashed arrows indicate the optic disc margins on the horizontal tomogram corresponding to that on the deviation map. T, temporal; N, nasal. (B-D) Time courses of changes in RA (B), DA (C) and CupV (D). Error bars show s.e.m. (*n*=3).

Fig. 2A indicates the regions measured for analysis of optical nerve head (ONH) parameters. No statistically significant effects were found in the saline group for all ONH parameters throughout the observation (*P*>0.05). Small changes in ONH parameters, including rim area (RA, mm^2^) and disc area (DA, mm^2^), were seen in the NMDA group, of which only DA showed a significant difference on day 30 compared to the baseline, but these parameters were not statistically different at day 60 (Fig. 2B,C). In the NMDA-injected eyes, there were no significant differences in cup volume (CupV, mm^3^) at any of the time points (Fig. 2D, *P*>0.05).

### Changes in thicknesses in the macular region

**Fig. 3.**
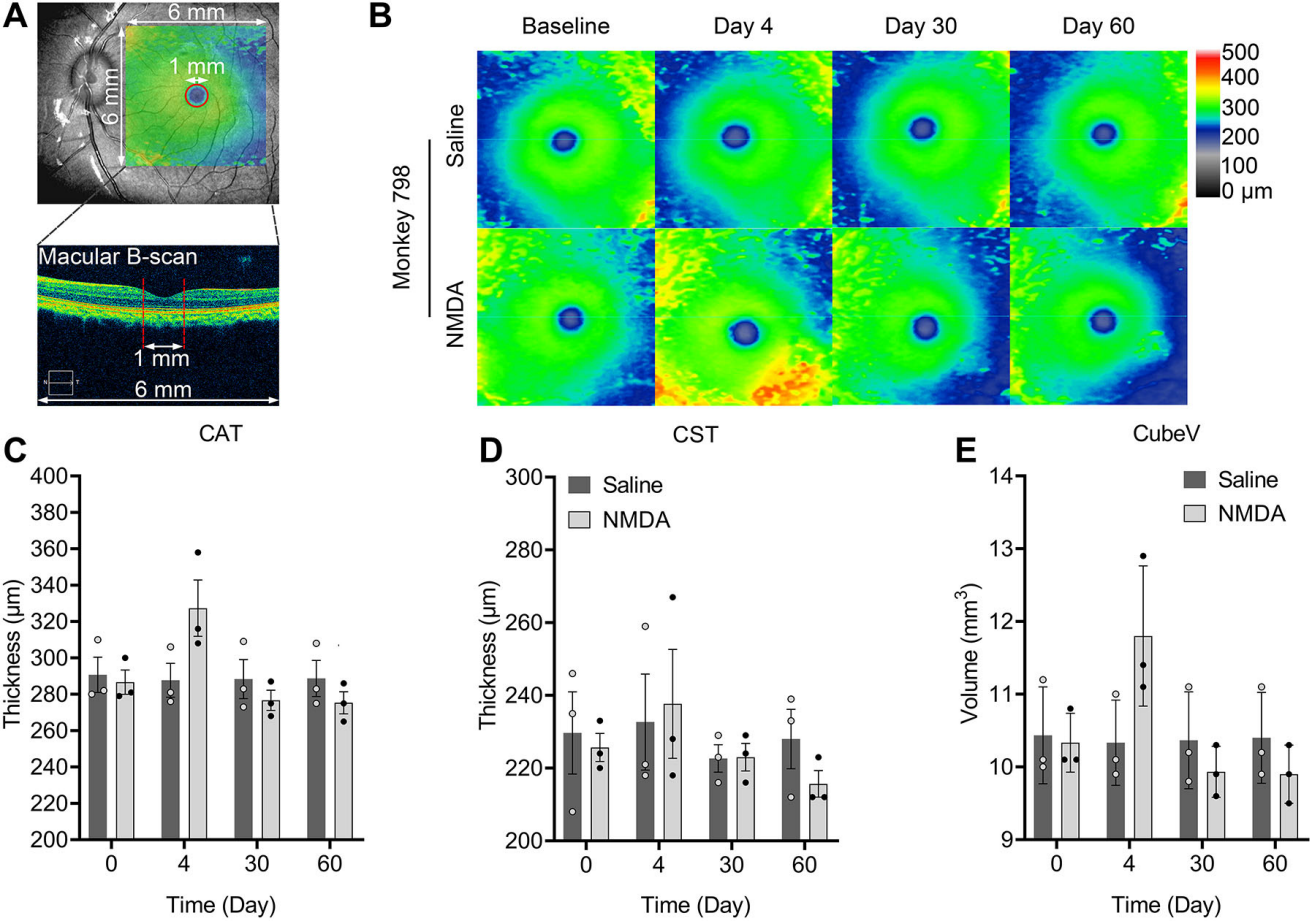
**Changes to macular parameters after intravitreal injection of NMDA.** Monkeys 312, 012 and 798 were used for these analyses. (A) Schematic diagram showing the analysis of relative parameters on the macular region (6×6 mm). N, nasal; T, temporal. (B) Representative maps of thickness from the inner limiting membrane to the retinal pigment epithelium layer (ILM-RPE) on monkey 798. (C-E) Time courses of changes in cube average thickness (CAT) (C), central subfield thickness (CST) (D) and cube volume (CubeV) (E). Error bars show s.e.m. (*n*=3).

Fig. 3A shows the region measured for analysis of macular thickness, which is presented in three different manners. Cube average thickness (CAT, μm) was measured as the total retinal thickness from the inner limiting membrane to the retinal pigment epithelium layer (ILM-RPE) in the 6×6 mm macular scan. The ILM-RPE thickness heat maps for monkey 798 at different time points are shown in Fig. 3B. The results for saline control- and NMDA-injected eyes are arranged in the upper and lower panels, respectively. No significant changes in ILM-RPE thickness were detected between the two groups. Cube volume (CubeV) represents the volume of the same region. Central subfield thickness (CST, μm) was measured as the ILM-RPE thickness of a circular region with a diameter of 1 mm in the central location of the 6×6 mm macular scan.

Saline injection did not produce any statistically significant effects for all macular parameters throughout the observation (*P*>0.05). The CAT, CST and CubeV showed the same changes as RNFL and GCA, which all increased on day 4 and then decreased gradually (Fig. 3C-E).

### Regional changes of the inner retina seen by cross-sectional scans

**Fig. 4.**
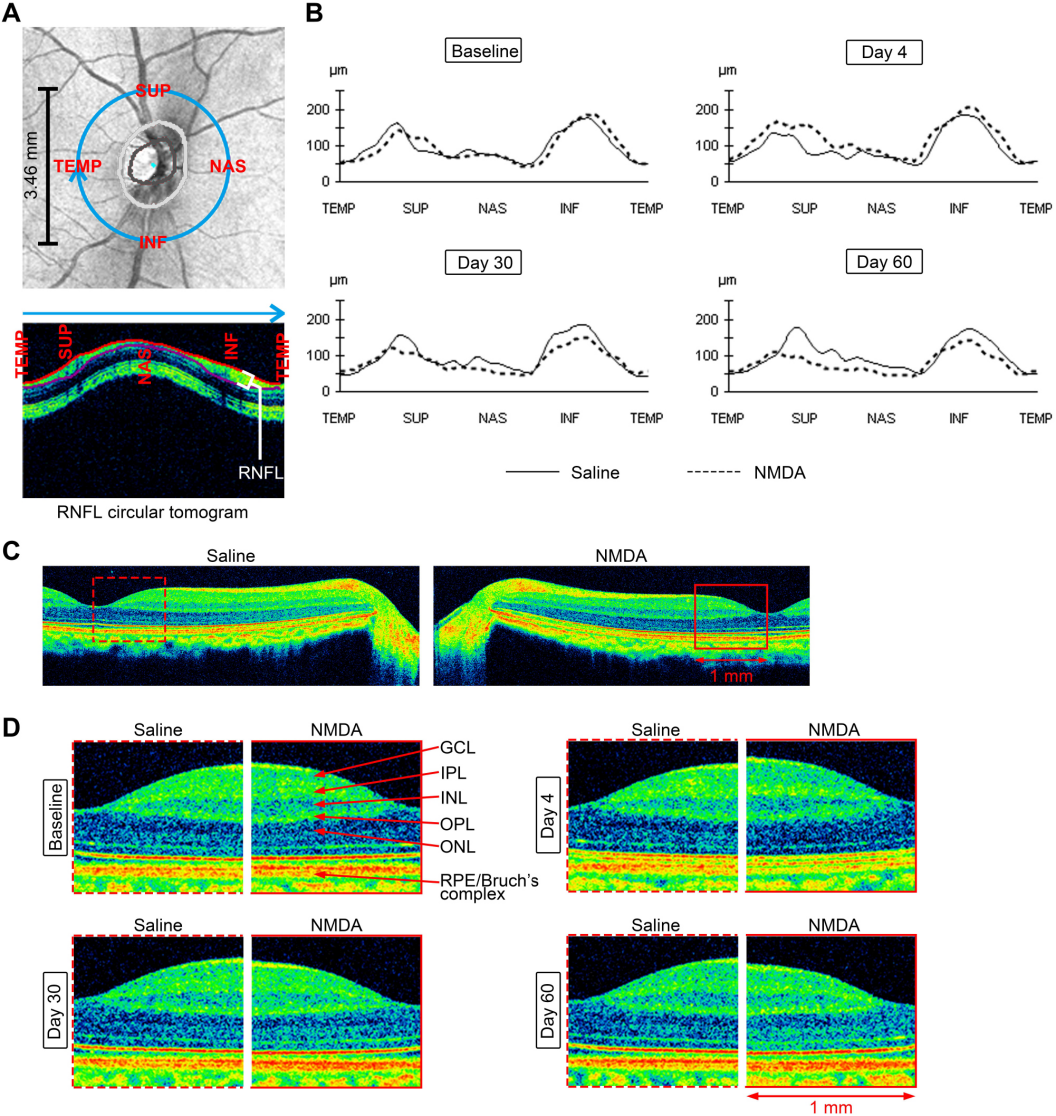
**Cross-sectional retinal images in the circum-papillary retina and the retina passing through the disc and fovea.** (A,B) Schematic diagram and circum-papillary retinal tomograms (A) and corresponding RNFL thickness profile (B) from monkey 798 after intravitreal injection of NMDA. The light blue circle (A, top) marks circular RNFL thickness around the optic disc, which was projected as a long strip in the order of temporal (TEMP), superior (SUP), nasal (NAS) and inferior (INF) quarters. The light gray, dark gray and purple lines in A represent the optic disc margin, the optic cup margin and the inner edge of the RNFL, respectively. (C) Schematic diagram for thickness alignment from high-definition (HD) line raster images from monkey 798. (D) Fovea thickness alignment. The regions of the nasal macular retina (1×1 mm) as shown in C were enlarged and marked by dashed red boxes in the saline-injected eye or by solid red boxes in the NMDA-injected eye. Scale bar: 1 mm. GCL, ganglion cell layer; IPL, inner plexiform layer; INL, inner nuclear layer; OPL, outer plexiform layer; ONL, outer nuclear layer; RPE, retinal pigment epithelium.

To obtain an initial understanding of the regional changes in retinal thickness, we performed and analyzed circum-papillary retinal scan of one monkey (#798). The top panel of Fig. 4A indicates the location of the circular circum-papillary RNFL scan with a 3.46 mm diameter from the center of optic disc. The retinas treated with NMDA exhibited RNFL thickening at day 4, and RNFL thinning at days 30 and 60 (Fig. 4B,D). This change corresponded to the results of mean RNFL analysis and was most pronounced in the superior, nasal and inferior quarters (Fig. 4B).

High-definition (HD) line raster scanning was used to capture a high-resolution cross-sectional image of the retina along a 6 mm line passing through the central part of the disc and fovea (Fig. 4C). As shown in Fig. 4B, the temporal direction of RNFL did not show thickness changes, and we mainly analyzed the changes in macular retinal morphology in an area of 1×1 mm. As showed in the enlarged views in Fig. 4D, in the NMDA-injected eyes, the thickness of the inner retina, such as the GCL and IPL, increased at first and then decreased. No morphological changes were observed in the saline-injected eyes.

### Electroretinography

Corresponding to the morphological changes, dysfunction of the RGCs was detected in electrophysiological studies [photopic negative response (PhNR) and pattern electroretinography (pERG)]. Interestingly, these changes preceded the measurable thinning of the GCL.

**Fig. 5.**
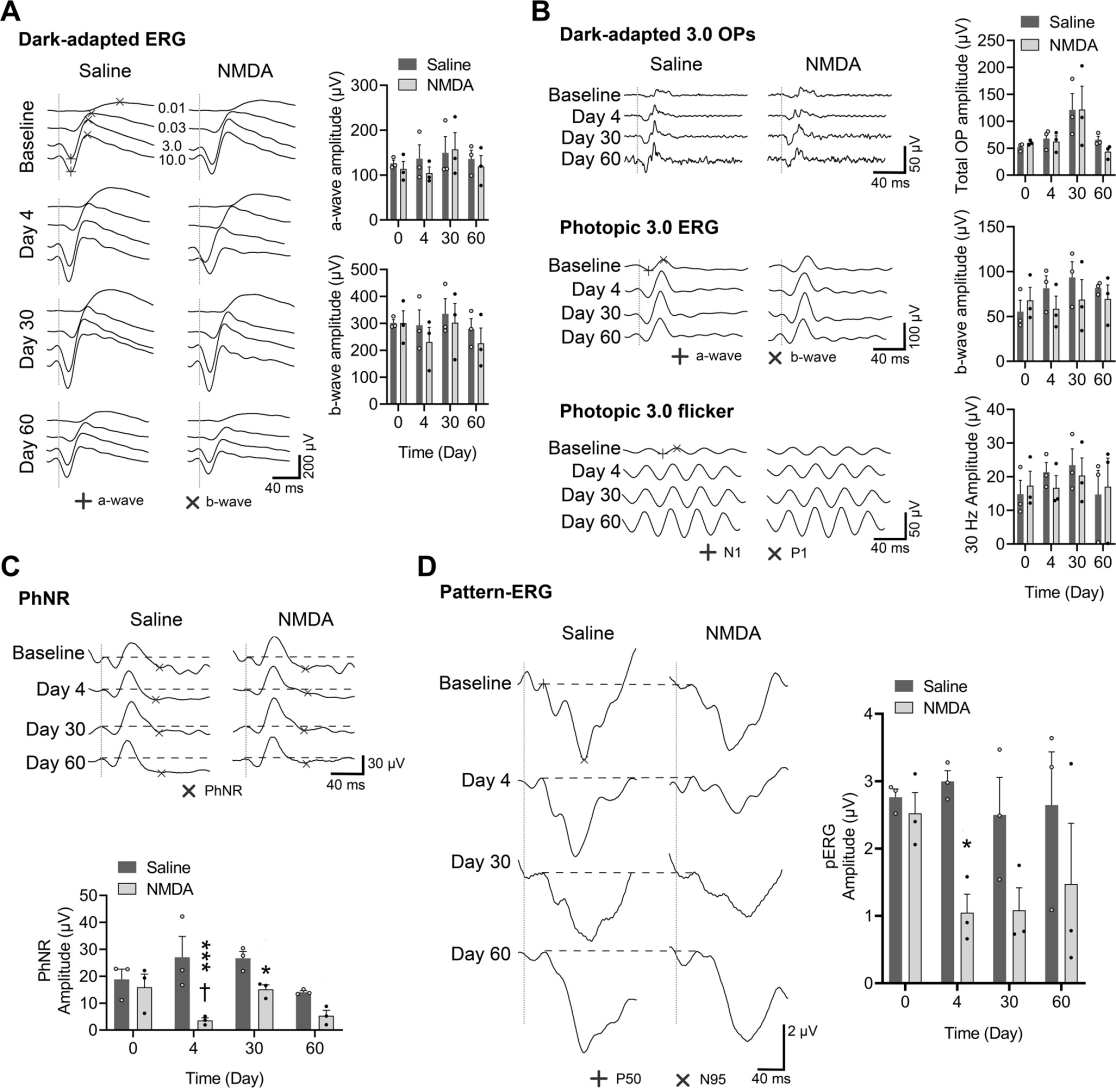
**Electroretinography responses of the NMDA-induced monkey retinal injury model.** (A) Representative dark-adapted full-field electroretinography (fERG) waves are shown on the left; a-and b-waves are marked as ‘+’ and ‘×’, respectively, and numbers between two waveform graphs show the corresponding stimulating light intensity. Dark-adapted 3.0 ERG statistical analyses are shown on the right. (B) Representative dark-adapted 3.0 oscillatory potential (OPs), photopic 3.0 ERG and flicker waveforms are shown on the left. The ‘+’ and ‘×’ symbols represent a- and b-waves in the photopic 3.0 ERG, and N1 and P1 amplitudes in the flicker response, respectively. Statistical analyses are shown on the right. (C) Representative ERG waves corresponding to phototopic negative response (PhNR) evaluation are shown on the top, and related statistical analyses are shown on the bottom. ‘×’ represents PhNR amplitudes. Using repeated-measures two-way ANOVA, interaction between groups and time course (*F*=2.109, *P*=0.1525), time factor (*F*=2.469, *P*=0.1121) and subjects factor (*F*=0.1946, *P*=0.9365) did not show significant difference, but the group factor was significant (*F*=78.72, *P*=0.0009). (D) Representative pattern electroretinography (pERG) scans are shown on the left and related statistical analysis on the right. ‘+’ and ‘×’ represent P50 and N95 amplitudes, respectively. Repeated-measures two-way ANOVA detected significance in the group factor of pERG (*F*=34.54, *P*=0.0277), but no statistical difference was detected in the time factors (*F*=0.9934, *P*=0.4572), subjects factor (*F*=3.071, *P*=0.0588) and interaction between groups and time course (*F*=3.38, *P*=0.0953). All vertical dotted lines indicate the onsets of stimuli (flash). In all statistical graphs, open and solid symbols indicate the corresponding values of control and NMDA-treated groups, respectively, for each monkey. Bars show the s.e.m. (*n*=3). ***P<0.001 and *P<0.05 indicate wave amplitudes dropping significantly after NMDA treatment (repeated measures two-way ANOVA analysis).

Fig. 5A,B shows that both saline and NMDA intravitreal injections affected scotopic and flicker wave amplitudes more or less, but no significant difference was detected in either the a- and b-waves in both scotopic and photopic full-field electroretinography (fERG), or the flicker and oscillatory potentials.

In contrast, the PhNR results (Fig. 5C) were different from the fERG results. On day 4, the PhNR amplitude of the NMDA-injected group dropped to 6.34±1.43 μV from 15.91±4.90 μV of the baseline. Repeated-measures two-way ANOVA analysis showed a significant effect of group (*F*=78.72, *P*=0.0009), but not of time (*F*=2.469, *P*=0.1121), subjects factor (*F*=0.1946, *P*=0.9365) or the interaction between group and time (*F*=2.109, *P*=0.1525), indicating that NMDA caused the change in PhNR from the beginning to the end of the study. Because the group factor of PhNR was significant, post hoc analysis was conducted. On days 4 and 30, the PhNR was significantly different between the NMDA-treated group and the corresponding control group by Fisher's LSD post hoc test (*P*=0.0004 and 0.0458, respectively).

Similarly, the results of pERG (Fig. 5D) also indicated that NMDA damaged the conducting function of the retina. On day 4, the pERG P50-N95 amplitudes of the NMDA-treated group decreased to 1.04±0.27 μV from 2.52±0.31 μV of the baseline, and it stayed low until day 60. Repeated-measures two-way ANOVA revealed a significant effect of group (*F*=34.54, *P*=0.0277), whereas the effects of time (*F*=0.9934, *P*=0.4572), subjects factor (*F*=3.071, *P*=0.0588) and the interaction between group and time (*F*=3.38, *P*=0.0953) were not significant. Because the group factor was significant, post hoc analysis was conducted as well. On day 4, a significant difference between the NMDA and control groups was detected by Fisher's LSD post hoc test (*P*=0.0154).

## DISCUSSION

As an excitatory neurotransmitter, NMDA activates neuronal signaling transductions, but activation of extrasynaptic NMDA receptors causes neurodegeneration and cell death (Kaur et al., 2012). Therefore, excessive extracellular NMDA in glaucoma is tightly related to RGC apoptosis, by stimulation of NMDA receptors (Hof et al., 1998; Ivanova et al., 2020; Kaur et al., 2012). NMDA-induced experimental retinal damage is well studied in rodents (Liu et al., 2022; Youale et al., 2022) and rabbits (Kinoshita et al., 2020), and has been used as a study model for retinal diseases, such as glaucoma, retinal ischemia and diabetic retinopathy (Niwa et al., 2016). However, as rodent and rabbit NMDA models lack ocular structures that humans have, such as the macula, it is difficult for them to mimic accurate NMDA-induced pathogenic processes in humans. Therefore, nonhuman primate models are irreplaceable for research on certain ophthalmic diseases such as macular dystrophies, glaucoma and age-related macular degeneration (Winkler et al., 2020). As a translational bridge between preclinical studies in rodent models and human clinical studies, nonhuman primate models are critically important in medication development (Phillips et al., 2014; Shen, 2010; Zhang et al., 2014). Careful and detailed characterization of nonhuman primate disease models is crucial in the development and meaningful application of these animal models (Mitchell et al., 2021). In this study, we evaluated the changes induced by NMDA intravitreal injection in rhesus monkeys.

The results of our study indicated similar specific damage to RGCs after NMDA injection, both structurally and functionally. OCT examination showed that both optic discs and macular areas of the NMDA-injected eyes presented with a transient thickening first, which was then followed by retinal thinning, compared to these regions in control eyes. The amplitudes of both PhNR and pERG decreased significantly and remained low until the end of the study. However, both a- and b-wave amplitudes of fERG did not show any significant changes compared to those of the control group.

RNFL thinning in both the ONH and macula, as well as GCL+IPL thinning in the macula, have been clinically detected to be associated with progressive worsening of glaucomatous damage (Christopher et al., 2018). In our study, the results of circum-papillary and macular RNFL analyses, macular GCA (Fig. 1) as well as HD five-line raster scanning (Fig. 4) showed an initial thickening of these structures at day 4 after NMDA injection, followed by a continuous and sustained thinning lasting 60 days. The superior and inferior areas of the circum-papillary retina displayed the biggest changes (Fig. 4A). However, areas besides the ONH and macula did not change significantly. These results indicate that NMDA may cause acute thickening at the ONH and macula initially, followed by continuous and prolonged thinning of retina, corresponding to RGC loss or other retinal damage or both. This preference of thickness change may be due to the density of RGC distribution, which matched our retinal thickness heat map results (Lee et al., 2012; Muniz et al., 2014).

The NMDA-induced retinal damage monkey model is not a model of ocular hypertension. The ONH parameters such as rim area, disc area and cup volume were not significantly affected as seen in ocular hypertension models (Koylu et al., 2021; Shimazawa et al., 2006). This model only mimicked glaucomatous neuropathy but not ocular hypertensive lesion.

In this study, unlike OCT changes that showed a process of thickening first and thinning last, all the electroretinography (ERG) wave amplitudes decreased by different degrees. The amplitudes of PhNR and pERG waves dropped significantly. However, no significant difference was detected in fERG. This may be because of the characteristics of different kinds of ERG examination and the specific manner of neuropathy induced by NMDA, which matches the OCT results as described below.

Dark-adapted (DA) fERG provides the comprehensive electrical response of the retina after light stimulation, which is mostly used to evaluate retinal function in a clinical setting. The initial negative a-wave in DA 3.0 and 10.0 ERGs (simulated at 3.0 and 10.0 cd s/m^2^ intensity, respectively) represents mixed rod and cone system responses (Robson et al., 2022). The positive b-wave arises largely in the rod-driven ON bipolar cells (Robson et al., 2022). Four to five small high-frequency rhythmic wavelets called oscillatory potentials are mainly derived from amacrine cells and other inner retinal tissues. In recent years, oscillatory potentials have been considered as sensitive electrophysiological indicators reflecting retinal ischemia and microvascular changes (Liao et al., 2023). Light-adapted fERG and flicker reflect the condition of steady-state cone cells (Tomczewski et al., 2024). In recent years, it has been generally believed that fERG did not include a measurable contribution from RGCs and it was rarely used in the diagnosis of optic nerve diseases (Robson et al., 2022). In this study, the a-wave was observed to have no significant changes, indicating that NMDA-induced damage in the retina had no injury on the function of photoreceptor cells. The b-wave was observed to be slightly lower than that of controls at days 4 and 60, but it was not statistically significantly different compared to the baseline and that at day 30, which indicated that NMDA-induced damage does not involve photoreceptor cells, bipolar cells or Müller cells, and that the changes might originate from the inner retina that interacts with cells in the outer retina. No significant changes were observed in tendency and amplitude of scotopic 3.0 oscillatory potentials, photopic 3.0 ERG and photopic 3.0 flicker, reflecting that amacrine cells can work normally in the retina. The result further revealed that the monkey model of NMDA-induced experimental retinal damage showed high specificity for damage to the inner retina.

pERG waves are generally accepted to originate from the GCL, and pERG is a sensitive method that could detect the damage to visual function caused by degeneration and apoptosis of ganglion cells and their axons. The typical change in pERG after RGC injury was reported to be a decrease in amplitude and a slower recovery rate (Holder, 2001; Porciatti et al., 2007). The abnormality of pERG was found to be a typical RGC damage signal. A late negative wave called N95 was the most sensitive component of pERG to optic neuropathy (Holder, 1987; Salgarello et al., 2021). In the current study, the continuous low amplitude of P50-N95 lasting until day 60 after NMDA intravitreal injection strongly indicates that NMDA damaged the function of RGCs.

Similar to pERG, which represents the conductive activity of RGCs, PhNR also arises from ganglion cells and their axons. PhNR is one of the ERG waveform components, such as a- and b-waves and oscillatory potentials, that is presented as a negative-going wave following the b-wave of the photopic ERG. Recently, PhNR has been found to be generated from the same source of the transient pERG N95 wave. Because of the large amplitude that was five to ten times higher than that of the typical N95 wave of pERG, PhNR could also specifically reflect the damage of RGCs and was easier to observe and measure than pERG (Cvenkel et al., 2022; Rangaswamy et al., 2004). Clinically, the reduction of focal PhNR amplitude in patients with primary open-angle glaucoma was associated with a local loss in retinal function in glaucoma, which had high sensitivity and specificity (Machida et al., 2008). In this study, the PhNR amplitude of eyes from the NMDA-induced damage group was significantly lower than that of their respective controls on day 4 after NMDA intravitreal injection, and the significant differences from the controls were maintained until days 30 and 60, although the amplitude increased slightly. The result was similar to the pERG wave, which further supports the specific induction of RGC injury by NMDA.

What is important is that, to our knowledge, NMDA-induced retinal thickening of the ONH and macular has not been previously reported in rodent NMDA models, even though they have been extensively studied (Chidlow et al., 2011; Fu and Sretavan, 2012; Luan et al., 2006). In rodents, the only report of ONH edema that we could find is a non-arteritic anterior ischemic optic neuropathy rat model, which is very different from a chemically induced retinal neuropathy model such as the NMDA-induced model (Luan et al., 2006). In rabbits, similar NMDA-induced retinal thickening was also detected by OCT and other means (Kinoshita et al., 2020). However, NMDA-induced retinal thickening is different from edema, because NMDA-injected eyes showed retinal thickening in the inner retina (IPL+GCL) transiently (Fig. 4D), which was different from common macula edema in the outer retina (Das et al., 2018). ONH and macular edemas do exist in clinical glaucoma cases. For instance, edema of the ONH is a well-documented observation of elevated intracranial pressure in glaucomatous eyes (McCulley et al., 2015). Murata et al. (2016) reported that microcystic macular edema is present in a number of patients with glaucoma and is associated with worse visual field mean deviation, pattern standard deviation and visual acuity. The NMDA-induced retinal thickness change may have a different mechanism of action from that of common retina edema, but both of them led to substantial retinal damage in patients. However, the clinical significance of edema and the subsequent disease progression and other related changes are unfortunately poorly investigated, such that more details await further studies. Therefore, the rhesus monkey model of NMDA-induced retinal damage may serve as a useful animal model for the pathophysiology and perhaps therapy of some of these retinopathies.

## MATERIALS AND METHODS

### Animals

Four male adult rhesus monkeys (*Macaca mulatta,* numbered as 012, 312, 798 and 896), which were raised in the Institute of Laboratory Animal Sciences, Sichuan Academy of Medical Sciences and Sichuan Provincial People's Hospital, were used in this study. All experiments were approved by the Ethics Committee of the Sichuan Provincial People's Hospital and conformed to the Association for Research in Vision and Ophthalmology (ARVO) Statement for the Use of Animals in Ophthalmic and Vision Research. Identity chips were subcutaneously implanted in each monkey at least 24 months before the initiation of this study. Animals were kept in individual, spacious, dedicated monkey cages, housed in a 22°C thermostatic room with a 12 h/12 h light/dark cycle. Sterilized food was provided three times a day following a veterinarian-designed meal plan, and clean drinking water was provided *ad libitum*.

One eye of each monkey was randomly selected for NMDA intravitreal injection, whereas the contralateral eye was assigned as a negative saline control receiving the same volume of saline intravitreal injection. Details are shown in Table 1. General anesthesia was achieved with intramuscular injection of Zoletil 50 (Virbac Lab, Carros, France) at a dosage of 10 mg/kg body weight before each ocular injection or examination.

**Table 1.**
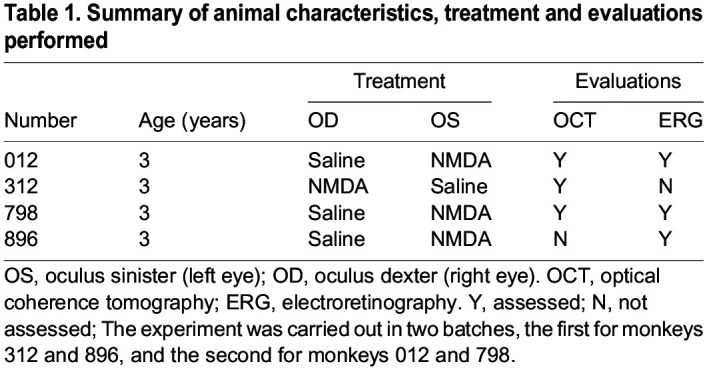
Summary of animal characteristics, treatment and evaluations performed

### Intravitreal injection

Anesthetized monkeys were placed supine under an ophthalmic operating microscope (Leica, Heerbrugg, Switzerland). After disinfecting the periorbital area, one drop of 0.5% proparacaine hydrochloride (Alcaine; Alcon Laboratories, Fort Worth, TX, USA) was applied to each cornea for topical anesthetic purpose, and one drop of 0.5% tropicamide phenylephrine (Mydrin P; Santen, Tokyo, Japan) was used to dilate the pupil. NMDA solution (105 μl, 0.2 mol/l NMDA in 0.9% NaCl; T6608, TargetMol Chemicals, Boston, MA, USA) or saline solution (105 μl, 0.9% NaCl) was slowly delivered to the vitreous chamber by a sterilized Hamilton glass micro-syringe with a 30G needle (Hamilton, Reno, NV, USA) under the ophthalmic operating microscope.

The chosen dose of NMDA solution was based on the vitreous volumes of monkeys (Rangaswamy et al., 2004) and previous NMDA studies (Kinoshita et al., 2020; Rangaswamy et al., 2004). The final concentration of NMDA in the vitreous cavity was chosen at a dose of 10 mM, which is slightly higher than that in previous studies, making it easier to observe more significant changes in retinal morphology.

The entry site was chosen 5 mm posterior to limbus on the temporal side. The needle was advanced until its tip was observed to be above the ONH and it was held still to avoid contact with any other intraocular tissue. Then, the drug or control solution was injected gently and slowly. The needle was left in the vitreous chamber for 2 min after the delivery and withdrawn quickly to minimize leakage. In order to prevent ocular infection, ofloxacin ophthalmic ointment (Santen, Tokyo, Japan) was topically administered after injection.

### OCT imaging

A Cirrus HD-OCT model 5000 (Carl Zeiss Meditec, Dublin, CA, USA) was used in this study. On day 0 (baseline; prior to intravitreal injection), days 4, 30 and 60 post injection, OCT examinations were performed. Each anesthetized monkey was placed on a customized platform. Head movement was prevented by a chinrest. The eyelids were manually opened gently only at the time of scanning. Optic disc cube 200×200, macular cube 200×200 and HD five-line raster scanning were performed by an experienced investigator. Images that showed a signal strength ≥6 were used for analysis.

The circum-papillary and macular areas were chosen automatically by the OCT system for the RNFL thickness analysis and GCA, as showed in Fig. 1B. A circle with 3.46 mm diameter in a solid purple line and gray shading marks the circum-papillary region (Fig. 1C), and the region between the outer and inner elliptical dashed purple lines indicates the region of GCA analysis (Fig. 1D).

The ONH parameter analyses, such as rim area, disc area and cup volume were also made by the OCT system automatically. Purple, black and red lines in Fig. 2A represent the region of analysis, optic disc and optic cup, respectively.

Central subfield thickness (CST), cube average thickness (CAT) and cube volume (CubeV) of the macular area were obtained by the macular cube 200×200 B scan as shown in Fig. 3. The 6×6 mm square region of macular area and circular region with 1 mm diameter of central fovea area were automatically chosen by the OCT system as shown in Fig. 3A.

In order to exhibit the full view of the NMDA-induced RNFL thickness change around the optic disc, an RNFL circular tomogram analysis was performed on monkey 798 as shown in Fig. 4A. The light blue circle marks circular RNFL thickness around the optic disc, which was projected as a long strip in the order of temporal, superior, nasal and inferior quarters. The macroscopic macular thickness change of the same monkey, as shown in Fig. 4B, was analyzed by choosing the same 1×1 mm square areas marked by dashed or solid red lines on the macular edge and aligning them on the same benchmark of RPE/Bruch's complex using the data from HD five-line raster scan.

### ERG

ERG was performed by using a Roland electroretinogram recorder and Ganzfeld Q450 stimulator (Roland Consult, Heidelberger, Germany). Examinations were conducted following the same time schedule as OCT. At every time point, standard fERG, pERG and PhNR were conducted as described previously (Frishman et al., 2018; Robson et al., 2018). Briefly, in a dark room, after at least 1 h of dark adaptation, monkeys were anesthetized and restrained in a customized monkey chair, facing directly toward the stimulator or screen (pERG). Topical application of 0.5% tropicamide phenylephrine was used for mydriasis and sodium hyaluronate (Hialid; Santen) was used for keeping corneas moist. Three needle-like reference electrodes sterilized with 75% alcohol were placed subcutaneously: one in the middle of the forehead (ground electrode) and the others on each side of the outer canthus. Disposable contact lens recording electrodes were placed directly in front of the pupils.

DA (scotopic) fERG was performed first, with a series of 0.01, 0.03, 0.1, 3.0 and 10.0 cd s/m^2^ stimulating luminance used in order. Then, scotopic oscillatory potential was recorded under 3.0 cd s/m^2^ light stimulation. After that, a 10 min adaptation period to white light (25 cd/m^2^) was instituted before photopic response assessments. Photopic 3.0 ERG and photopic 3.0 flicker (30 Hz) responses were recorded under corresponding stimulations (3.0 cd s/m^2^ superimposed on a 30 cd/m^2^ background). According to the direction (Robson et al., 2018), a-wave amplitude was set to the negative wave trough, and the b-wave amplitude was measured from the a-wave trough to the next highest positive wave (b-wave) peak. Flicker ERG was picked as one of these average waves and measured from trough to peak of a typical wave.

Photopic ERGs for PhNR analysis were recorded later. A blue background (470 nm, 25 cd/m^2^) light adaptation lasting for 30 min was applied before PhNR initiation. Red flash stimulation at 625 nm wavelength, 3.0 cd s/m^2^ intensity, 150 ms plot time and 0.2 Hz frequency was used to evaluate the PhNR. Its amplitude was measured from baseline to the next negative trough right after the b-wave (PhNR).

pERG was not conducted until pupils had recovered from mydriasis. After 30 min of white-color 25 cd/m^2^ ambient light adaptation, pERG tests were started. A full-field stimulating screen was used. The stimulus frequency and plot time were set to 4.28 Hz and 180 ms, respectively. P50 to N95 wave amplitude was measured from the first peak (P50) to the lowest trough (N95).

### Statistics

GraphPad Prism 6 software (GraphPad software, Boston, MA, USA) was used for all statistics. The statistical significance was calculated using repeated-measures two-way ANOVA. Only when the effects of group, time or the interaction between group and time were significant, Fisher's LSD post hoc test was conducted. *P*<0.05 was considered significant. Data are shown as mean±standard error of the mean (s.e.m.).
